# Left ventricular trabecular complexity for risk stratification of cancer therapy–related cardiac dysfunction in breast cancer

**DOI:** 10.1002/mco2.70004

**Published:** 2025-01-02

**Authors:** Hesong Shen, Qian Xu, Chunrong Tu, Yangling Peng, Yuhang Xie, Zhiming Miao, Rui Yang, Jiuquan Zhang

**Affiliations:** ^1^ Department of Radiology Chongqing University Cancer Hospital & Chongqing Cancer Institute & Chongqing Cancer Hospital Chongqing China; ^2^ School of Medicine Chongqing University Chongqing China

**Keywords:** breast cancer, cardiotoxicity, magnetic resonance imaging

## Abstract

The left ventricular trabecular fractal dimension (LVTFD) derived from cardiac magnetic resonance reflects myocardial trabecular complexity, which is associated with cardiovascular disease risk. Baseline risk stratification of cancer therapy–related cardiac dysfunction (CTRCD) in patients with breast cancer who received anthracycline is a very important clinical issue. In this study, we used the Cox model to derive and validate a new score system based on LVTFD for baseline risk stratification of CTRCD in breast cancer patients receiving anthracycline. We also compare the performance of LVTFD‐based score with the Heart Failure Association‐International Cardio‐Oncology Society (HFA‐ICOS) score using C‐index. This study enrolled 370 participants, of whom 73 participants developed CTRCD. The C‐indices of LVTFD‐based score integrating age, hypertension, previous cardiovascular disease, and maximal apical fractal dimension were higher than those of HFA‐ICOS score for stratifying CTRCD (0.834 vs. 0.642 and 0.834 vs. 0.633, respectively, in derivation and validation cohort). LVTFD‐based score can stratify the CTRCD risk, but HFA‐ICOS score cannot. The above results reveal that the LVTFD‐based score is an alternative method for baseline risk stratification of CTRCD in breast cancer who received anthracycline.

## Graphical abstract

The left ventricular trabecular fractal dimension (LVTFD)‐based score integrating age, hypertension, previous cardiovascular disease, and maximal apical FD outperforms Heart Failure Association‐International Cardio‐Oncology Society (HFA‐ICOS) score for stratifying cancer therapy–related cardiac dysfunction (CTRCD) in breast cancer patients receiving anthracycline.

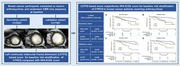

## 1 INTRODUCTION

Anthracyclines are often used in the treatment of breast cancer.^1^ Patients with breast cancer receiving anthracyclines are at high risk of cancer therapy–related cardiac dysfunction (CTRCD), which reduces adherence to anti‐cancer treatments and may ultimately decrease the overall survival rates.^2^ Therefore, risk stratification of CTRCD before anthracycline chemotherapy is important for breast cancer patients.

Baseline risk stratification of CTRCD is a challenging task in breast cancer patients with anthracycline administration.^3^ These clinically known cardiovascular disease (CVD) risk factors (e.g., age, body mass index, diabetes, hypertension, dyslipidemia, and smoking) failed to accurately stratify CTRCD risk owing to ignoring the differences in cancer treatment modalities and cardiac structural and functional subtypes.^4^, ^5^ According to the 2022 European Society of Cardiology guidelines on cardio‐oncology, the Heart Failure Association‐International Cardio‐Oncology Society (HFA‐ICOS) score including previous CVDs, cardiac biomarkers, demographic and cardiovascular risk factors, previous cardiotoxic cancer treatment, and lifestyle risk factors is recommended to baseline risk stratification of CTRCD in breast patients receiving anthracyclines.^6^ However, this recommendation is derived from level of evidence B or C.^7^ Therefore, validation of the current HFA‐ICOS score and new parameters that can predict risk of CTRCD are priorities in breast cancer patients receiving anthracyclines.

Cardiac magnetic resonance (CMR) cine imaging, which is a basic part of the CMR examination, has been established as a noninvasive and noncontrast modality for assessing the structure and function of the heart.^8^ Left ventricular ejection fraction (LVEF) and global longitudinal strain (GLS) derived from CMR cine images are considered as risk predictors of CTRCD in breast cancer patients receiving anthracyclines.^9^ However, the accuracy of LVEF and GLS at baseline for risk stratifying CTRCD remains controversial, because LVEF often identifies only irreversible CTRCD, and GLS is highly dependent on the load and chamber size of left ventricle.^10^, ^11^

The left ventricular trabecular fractal dimension (LVTFD) derived from CMR cine sequence reflects myocardial trabecular complexity and has been identified as a new and important parameter for the determinant of cardiac performance and has a causal relationship with the risk of CVD.^12^ Recent studies have reported that LVTFD is a new biomarker of cardiac involvement in Fabry disease^13^ and risk stratification of adverse cardiovascular events in hypertrophic cardiomyopathy.^14^ However, it is unclear whether LVTFD can be used as a new parameter to risk stratification of CTRCD and whether it improves the performance of HFA‐ICOS score for risk stratification of CTRCD in breast cancer patients receiving anthracyclines.

Therefore, this study aimed to (i) validate the current HFA‐ICOS score for baseline risk stratification of CTRCD and (ii) derive and validate an LVTFD‐based score for stratifying CTRCD risk in comparison to HFA‐ICOS score in breast cancer patients receiving anthracyclines.

## 2 RESULTS

### 2.1 Study population characteristics

**FIGURE 1:**
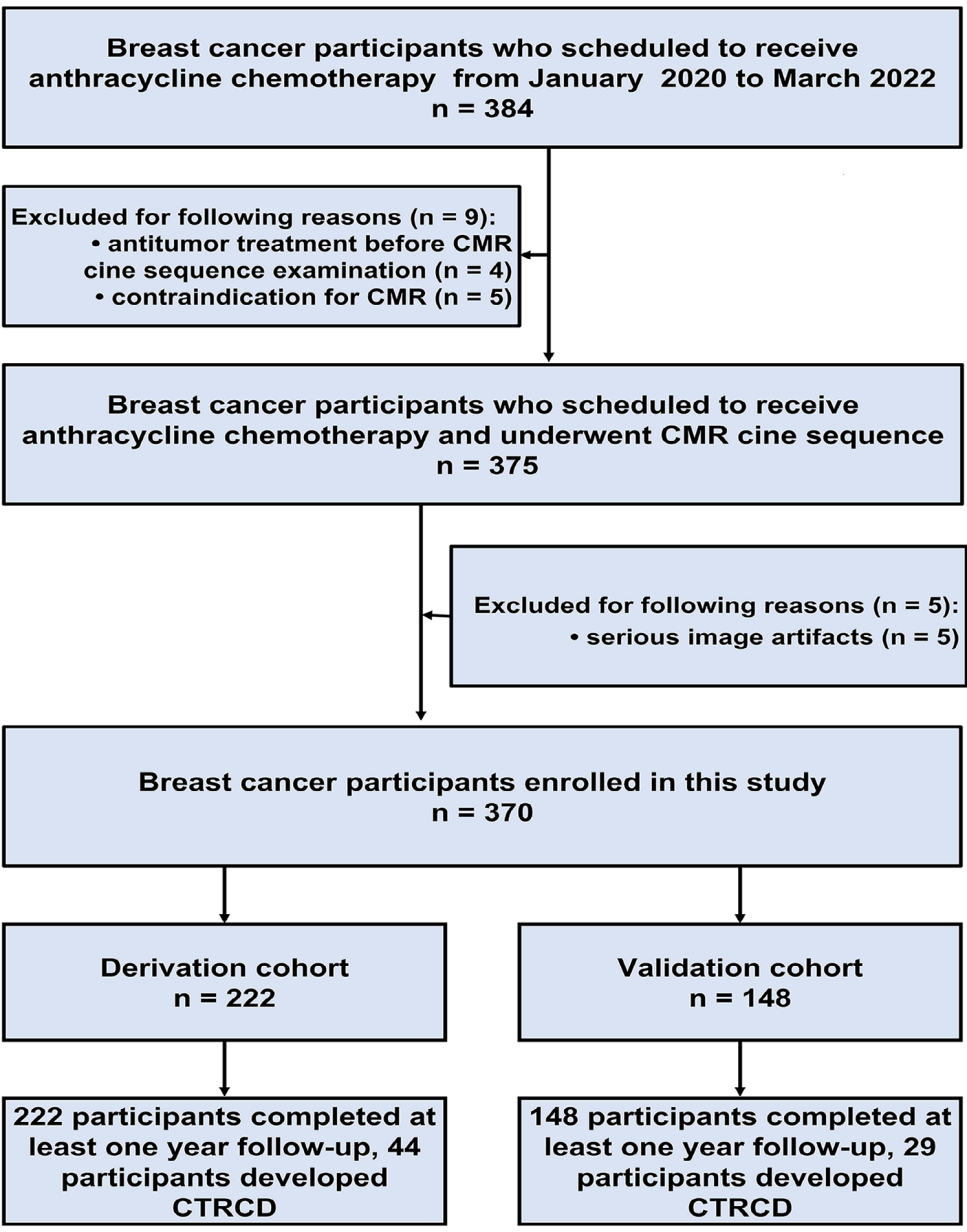
Flowchart of this study. CMR, cardiac magnetic resonance; CTRCD, cancer therapy–related cardiac dysfunction.

The flowchart of this study is shown in Figure 1. Fourteen participants were excluded because of anti‐tumor treatment before CMR (*n* = 4), contraindication to magnetic resonance imaging (*n* = 5), and serious artifacts of CMR (*n* = 5). Finally, 370 participants were enrolled and they were divided into the derivation cohort (*n* = 222) and the validation cohort (*n* = 148). CTRCD occurred in 73 (73/370, 19.8%) participants (44 [44/222, 20.0%] in derivation cohort and 29 [29/148, 19.6%] in validation cohort). The median CTRCD‐free survival time was 18.0 [interquartile range (IQR), 12–24] months.

The demographic and clinical characteristics and CMR data are shown in Table 1. There were no differences in baseline demographic and clinical characteristics except for hypertension and previous CVD between those with and without CTRCD in both derivation and validation cohorts.

**TABLE 1:** Baseline demographic, clinical, and CMR data in derivation and validation cohorts.

	Derivation cohort (*N* = 222)			Validation cohort (*N* = 148)		
Variable	Without CTRCD (*N* = 178)	With CTRCD (*N* = 44)	*p*	Without CTRCD (*N* = 119)	With CTRCD (*N* = 29)	*p*
Age at cancer diagnosis, years old			0.10			0.16
≥80	0 (0)	0 (0)		0 (0)	0 (0)	
65–79	`14 (7.9)	7 (15.9)		`10 (8.4)	5 (17.2)	
<65	184 (92.6)	37 (91.9)		109 (91.6)	34 (86.2)	
Molecular subtype						0.46
Luminal	116 (65.2%)	25 (56.8%)	0.55	78 (65.5%)	16 (55.2%)	
TNBC	29 (16.3%)	8 (18.2%)		20 (16.8%)	5 (17.2%)	
HER2‐enriched	33 (18.5%)	11 (25.0%)		21 (17.6%)	8 (27.6%)	
Clinical stage			0.93			0.82
I	27 (15.2%)	7 (15.9%)		18 (15.1%)	6 (20.7%)	
II	63 (35.4%)	15 (34.1%)		41 (34.4%)	8 (27.6%)	
III	80 (44.9%)	19 (43.2%)		54 (45.4%)	13 (44.8%)	
IV	8 (4.5%)	3 (6.8%)		6 (5.0%)	2 (6.9%)	
Diabetes, *n* (%)	8 (4.5)	5 (11.4)	0.082	6 (5.0)	3 (10.3)	0.28
Hypertension, *n* (%)	16 (9.0)	9 (20.5)	0.031	9 (7.6)	6 (20.7)	0.036
Hyperlipidemia, *n* (%)	23 (12.9)	8 (18.2)	0.37	14 (11.8)	5 (17.2)	0.43
Chronic kidney disease, *n* (%)	0 (0)	0 (0)	1.00	0 (0)	0 (0)	1.00
Current smoker or significant smoking history (%)	6 (4.1)	2 (5.4)	0.72	5 (4.2)	2 (6.9)	0.54
BMI (kg/m^2^)			0.40			0.28
≥30	10 (5.6)	4 (9.1)		6 (5.0)	3 (10.3)	
<30	141 (95.3)	34 (91.9)		113 (95.0)	26 (89.7)	
Elevated baseline cTn or NP, *n* (%)	2 (1.1)	2 (4.5)	0.13	2 (1.7)	1 (3.4)	0.55
Previous cardiovascular disease			0.002			0.002
Heart failure/cardiomyopathy	0 (0)	0 (0)		0 (0)	0 (0)	
Severe valvular heart disease	0 (0)	0 (0)		0 (0)	0 (0)	
MI or PCI or CABG	0 (0)	0 (0)		0 (0)	0 (0)	
Stable angina	6 (3.4)	7 (15.9)		3 (2.5)	5 (17.2)	
Cardiac medications						
ACE inhibitors	5 (2.8)	2 (4.5)	0.56	3 (2.5)	1 (3.4)	0.78
Angiotensin receptor blocker	3 (1.7)	1 (2.3)	0.79	2 (1.7)	1 (3.4)	0.55
Beta blocker	6 (3.4)	2 (4.5)	0.71	3 (2.5)	1 (3.4)	0.78
Statins	5 (2.8)	3 (6.8)	0.20	4 (3.4)	2 (6.9)	0.39
Therapeutic regimen			0.77			0.67
TAC, *n* (%)	71 (40.0%)	17 (38.2)		48 (40.7%)	11 (36.9%)	
AC‐T, *n* (%)	63 (35.4%)	13 (30.0%)		42 (35.1%)	8 (28.5%)	
AT, *n* (%)	11 (6.1%)	3 (6.8%)		8 (6.6%)	2 (7.0%)	
AC‐THP, *n* (%)	33 (18.5%)	11 (25.0%)		21 (17.6%)	8 (27.6%)	0.23
CMR parameters						
LVEF (%)	65.83 ± 4.85	64.65 ± 7.02	0.30	66.21 ± 4.45	64.43 ± 7.26	0.22
LVEDV (mL)	97.20 ± 22.16	97.64 ± 10.20	0.85	96.83 ± 21.55	100.10 ± 8.46	0.20
LVESV (mL)	33.39 ± 9.21	35.09 ± 7.20	0.19	32.69 ± 9.08	35.87 ± 7.36	0.082
LVMASS (g)	74.72 ± 11.26	72.00 ± 11.96	0.16	74.72 ± 11.18	72.39 ± 11.07	0.32
GLS	18.59 ± 2.13	17.73 ± 3.41	0.12	18.732 ± 2.049	17.70 ± 3.36	0.12
GCS	23.24 ± 2.24	22.83 ± 2.02	0.10	22.29 ± 2.23	22.83 ± 2.03	0.24
GRS	32.99 ± 2.85	33.06 ± 2.99	0.89	33.11 ± 2.85	33.01 ± 3.12	0.87
Global FD	1.190 ± 0.019	1.222 ± 0.032	<0.001	1.190 ± 0.017	1.219 ± 0.028	<0.001
Maximal basal FD	1.234 ± 0.020	1.263 ± 0.034	<0.001	1.234 ± 0.018	1.260 ± 0.030	<0.001
Mean basal FD	1.178 ± 0.017	1.208 ± 0.031	<0.001	1.178 ± 0.019	1.206 ± 0.028	<0.001
Maximal apical FD	1.262 ± 0.022	1.291 ± 0.034	<0.001	1.262 ± 0.020	1.289 ± 0.029	<0.001
Mean apical FD	1.188 ± 0.018	1.217 ± 0.032	<0.001	1.188 ± 0.018	1.214 ± 0.029	<0.001

Abbreviations, ACE, angiotensin‐converting enzyme; AC‐T, doxorubicin + cyclophosphamide + docetaxel; AC‐THP, doxorubicin + cyclophosphamide + docetaxel + trastuzumab + pertuzumab; AT, doxorubicin + docetaxel; BMI, body mass index; CABG, coronary artery bypass graft; CMR, cardiac magnetic resonance; cTn, cardiac troponin; CTRCD, cancer therapy–related cardiac dysfunction; FD, fractal dimension; GCS, global circumferential strain; GLS, global longitudinal strain; GRS, global radial strain; HER2, human epidermal growth factor receptor 2; LVEDV, left ventricular end‐diastolic volume; LVEF, left ventricular ejection fraction; LVESV, left ventricular end‐systolic volume; LVMASS, left ventricular mass; MI, myocardial infarction; NP, natriuretic peptides; PCI, percutaneous coronary intervention; TNBC, triple negative breast cancer PCI; TAC, docetaxel + doxorubicin + cyclophosphamide.

There were excellent intra‐ and interobserver reproducibility in LVEF, left ventricular end‐diastolic volume (LVEDV), end‐systolic volume (LVESV), and myocardial mass (LVMASS), GLS, global circumferential strain (GCS), global radial strain (GRS), global fractal dimension (FD), maximal basal FD, mean basal FD, maximal apical FD, and mean apical FD, with the interclass correlation coefficient (ICC) value ranging from 0.846 to 0.957. Detailed ICC values for all variables are presented in Table S1. Pearson's correlation analysis showed that all left ventricular FDs were not associated with ventricular function and mass parameters, as shown in Tables S2 and S3.

In both derivation and validation cohort, the global FD, maximal and mean basal FD, and maximal and mean apical FD were higher in participants with CTRCD than those in participants without CTRCD (all *p* < 0.05). There were no differences in GLS, GCS, and GRS between those with and without CTRCD in both derivation and validation cohorts (all *p* > 0.05).

### 2.2 Derivation of LVTFD‐based score

**FIGURE 2:**
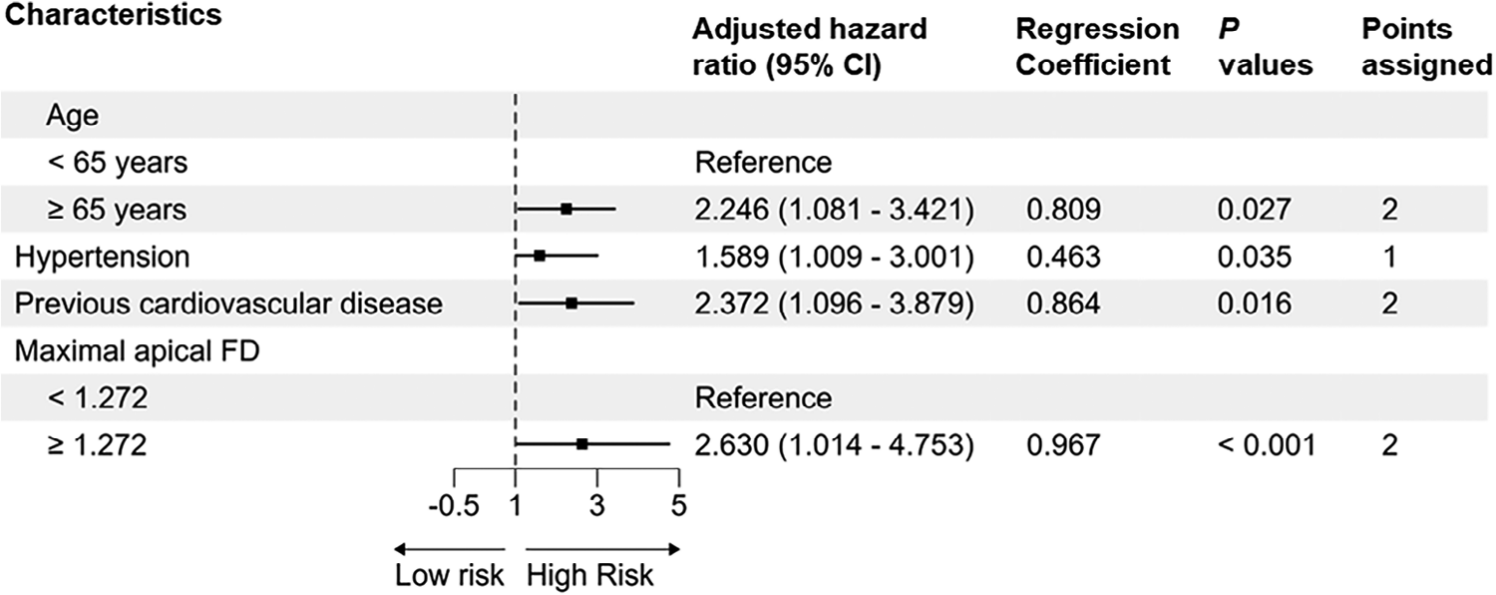
Hazard ratio, regression coefficients, and point assignment for each risk factor in multivariable Cox model. CI, confidence interval; FD, fractal dimension.

As shown in Figure 2, the age, hypertension, previous CVD, and maximal apical FD (adjusted hazard ratios: 2.246, 1.589, 2.372, and 2.630, respectively) were selected as components for the LVTFD‐based score. We considered a woman aged <65 years without hypertension or previous CVD and with maximal apical FD <1.272 as the reference, she would have a LVTFD‐based score of 0. Individual risk factors contributed 1 or 2 points to the LVTFD‐based score. Individuals with hypertension were assigned 1 point and individuals aged ≥65 years or those with previous CVD or maximal apical FD ≥ 1.272 were assigned 2 points. The subjects with LVTFD‐based scores of 0–1, 2–3, and ≥ 4 points were divided into low‐, moderate‐, and high‐risk groups, respectively.

### 2.3 Comparison of HFA‐ICOS score and LVTFD‐based score performance for the baseline risk stratification of CTRCD

As shown in Table 2, there were significant differences in HFA‐ICOS score and LVTFD‐based score between the participants with and without CTRCD in both derivation and validation cohorts (all *p* < 0.001). In derivation cohort, 84.3%, 11.8%, and 3.9% of participants without CTRCD were classified as low‐, moderate‐, and high‐risk and 50.0%, 31.8%, and 18.2% of participants with CTRCD were classified as low‐, moderate‐, and high‐risk according to the HFA‐ICOS score. In derivation cohort, 59.6%, 36.5%, and 3.9% of participants without CTRCD were classified as low‐, moderate‐, and high‐risk, and 27.3%, 50.0%, and 22.7% of participants with CTRCD were classified as low‐, moderate‐, and high‐risk according to the LVTFD‐based score. Similar results were found in validation cohort.

**TABLE 2:** The HFA‐ICOS score and LVTFD score in derivation and validation cohorts.

	Derivation cohort (*N* = 222)			Validation cohort (*N* = 148)		
Variable	Without CTRCD (*N* = 178)	With CTRCD (*N* = 44)	*p*	Without CTRCD (*N* = 119)	With CTRCD (*N* = 29)	*p*
HFA‐ICOS score			<0.001			<0.001
Low risk	150 (84.3)	22 (50.0)		102 (85.7)	12 (41.4)	
Moderate risk	21 (11.8)	14 (31.8)		13 (10.9)	12 (41.4)	
High risk	7 (3.9)	8 (18.2)		4 (3.4)	5 (17.2)	
Very high risk	0 (0)	0 (0)		0 (0)	0 (0)	
LVTFD‐based score			<0.001			<0.001
Low risk (0–1 point)	106 (59.6)	12 (27.3)		72 (60.5)	8 (27.6)	
Moderate risk (2–3 points)	65 (36.5)	22 (50.0)		42 (35.3)	24 (48.3)	
High risk (≥4 points)	7 (3.9)	10 (22.7)		5 (4.2)	7 (24.1)	

*Note*: LVTFD‐based score, the combination of age, hypertension, previous cardiovascular disease, and maximal apical FD by Cox model.

Abbreviations: CTRCD, cancer therapy–related cardiac dysfunction; HFA‐ICOS, Heart Failure Association–International Cardio‐Oncology Society; LVTFD, left ventricular trabecular fractal dimension.

As shown in Table 3, the C‐indices of LVTFD‐based score were higher than those of HFA‐ICOS score for the baseline risk stratification of CTRCD (0.834 vs. 0.642, *p *< 0.001 and 0.834 vs. 0.633, *p *< 0.001, respectively, in the derivation and validation cohorts).The C‐indices of LVTFD‐based score were higher than those of the combination of age, hypertension, and previous CVD (0.834 vs. 0.671, *p *< 0.001 and 0.834 vs. 0.674, *p* < 0.001, respectively, in both derivation and validation cohorts).

**TABLE 3:** The performance of these parameters for predicting CTRCD in derivation and validation cohort.

	C‐index (95% confidence interval)	*p*	p*	p**
Derivation cohort				
Age + hypertension + previous cardiovascular disease	0.671 (0.588–0.812)	<0.001	0.85	0.43
HFA‐ICOS score	0.642 (0.454–0.716)	<0.001	0.081	
Maximal apical FD (≥1.272)	0.688 (0.532–0.810)	<0.001		
LVTFD‐based score	0.834 (0.701–0.932)			
Validation cohort				
Age + hypertension + previous cardiovascular disease	0.664 (0.503–0.761)	<0.001	0.80	0.37
HFA‐ICOS score	0.633 (0.416–0.705)	<0.001	0.084	
Maximal apical FD (≥1.272)	0.679 (0.521–0.798)	<0.001		
LVTFD‐based score	0.830 (0.698–0.925)			

*Note*: LVTFD‐based score, the combination of age, hypertension, previous cardiovascular disease, and maximal apical FD by Cox model. *p*, LVTFD‐based score versus maximal apical FD or HFA‐ICOS score by Delong test; p*, maximal apical FD versus HFA‐ICOS score by Delong test; p**, HFA‐ICOS score versus age + hypertension + previous cardiovascular disease by Delong test.

Abbreviations: CTRCD, cancer therapy–related cardiac dysfunction; FD, fractal dimension; HFA‐ICOS, Heart Failure Association–International Cardio‐Oncology Society; LVTFD, left ventricular trabecular fractal dimension.

**FIGURE 3:**
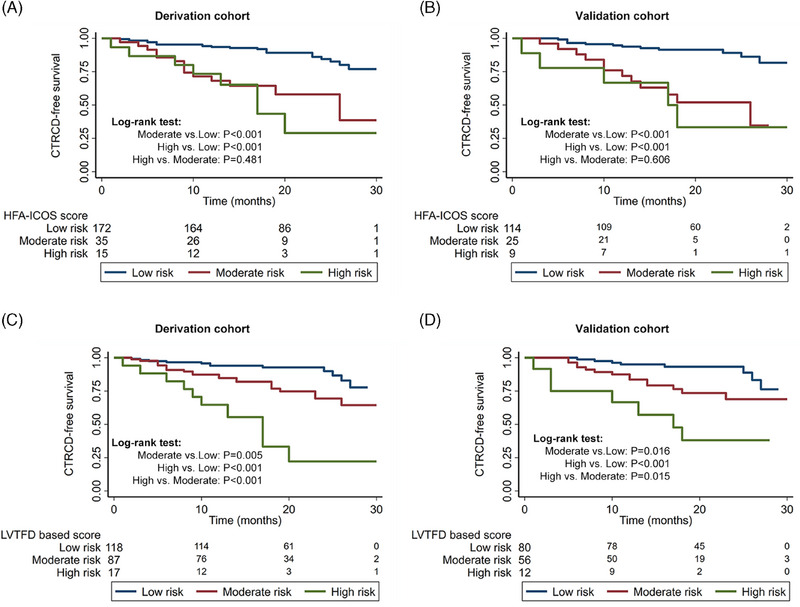
Kaplan‐Meier curves for CTRCD. HFA‐ICOS score failed to stratify the CTRCD between the high‐ and moderate‐risk subjects in derivation (A) and validation cohort (B). LVTFD‐based score successfully stratified the CTRCD among the high‐, moderate‐, and low‐risk subjects in derivation (C) and validation cohort (D). CTRCD, cancer therapy–related cardiac dysfunction; HAF‐ICOS, Heart Failure Association‐International Cardio‐Oncology Society; LVTFD, left ventricular trabecular fractal dimension.

Kaplan–Meier survival curves stratified by HFA‐ICOS score for the baseline risk stratification of CTRCD are shown in Figure 3. The CTRCD‐free survival of high‐risk subjects and moderate‐risk subjects was higher than low‐risk subjects (all *p* < 0.001), respectively. However, the CTRCD‐free survival showed no difference between high‐ and moderate‐risk subjects in both the derivation and validation cohorts (*p* = 0.481 and *p* = 0.606, respectively).

Kaplan–Meier survival curves analysis stratified by LVTFD‐based score for baseline risk stratification of CTRCD are shown in Figure 3. The CTRCD‐free survival of high‐risk subjects and moderate‐risk subjects was higher than that of low‐risk subjects, respectively, in both derivation (*p* < 0.001, *p* = 0.005) and validation cohorts (*p* < 0.001, *p* = 0.016). In addition, the CTRCD‐free survival of high‐risk subjects was also significantly higher than that of moderate‐risk subjects in both derivation (*p* < 0.001) and validation cohorts (*p* = 0.015).

The CTRCD rate at 1 year, 2 years, and the end of follow‐up of different risk subgroups are listed in Table 4.

**TABLE 4:** CTRCD rates at different time points stratified by the HFA‐ICOS score and LVTFD‐based score.

	CTRCD rates (derivation cohort)			CTRCD rates (validation cohort)		
Variable	At 1 year	At 2 years	At the end of follow‐up time	At 1 year	At 2 years	At the end of follow‐up time
HFA‐ICOS score						
Low risk	11/172 (6.4%)	19/172 (11.0%)	22/172 (12.8%)	7/114 (6.4%)	10/114 (8.8%)	12/114 (10.5%)
Moderate risk	11/35 (31.4%)	13/35 (34.3%)	14/35 (40.0%)	7/25 (28.0%)	11/25 (44.0%)	12/25 (48.0%)
High risk	4/15 (26.7%)	8/15 (53.3%)	8/15 (53.3%)	3/9 (33.3%)	5/9 (55.6%)	5/9 (55.6%)
Very high risk	0/0 (0%)	0/0 (0%)	0/0 (0%)	0/0 (0%)	0/0 (0%)	0/0 (0%)
LVTFD‐based score						
Low risk (0–1 point)	7/118 (5.9%)	9/118 (7.6%)	12/118 (10.1%)	4/80 (5.0%)	5/80 (6.3%)	8/80 (10.3%)
Moderate risk (2–3 points)	13/87 (14.9%)	21/87 (24.1%)	22/87 (25.3%)	9/56 (16.1%)	14/56 (25.0%)	14/56 (25.0%)
High risk (≥4 points)	6/17 (35.3%)	10/17 (58.8%)	10/17 (58.8%)	4/12(33.3%)	7/12 (58.3%)	7/12 (58.3%)

Abbreviations: CTRCD, cancer therapy–related cardiac dysfunction; HFA‐ICOS, Heart Failure Association–International Cardio‐Oncology Society; LVTFD, left ventricular trabecular fractal dimension.

## 3 DISCUSSION

In this study, we found that the performance of HFA‐ICOS score for baseline risk stratification of CTRCD was unsatisfactory. Our new LVTFD‐based score integrating clinical risk factors and CMR‐derived LVTFD significantly improved the performance of the baseline risk stratification for CTRCD.

Baseline risk stratification of CTRCD is important in patients with breast cancer receiving anthracycline, because it enables the oncologist to consider cardiotoxicity risk and make personalizing cancer treatment and cardioprotective strategies.^15^ The HFA‐ICOS score is considered to determine baseline risk of CTRCD in cancer patients receiving anthracyclines.^16^ Our study verified the performance of HFA‐ICOS score for baseline risk stratification of CTRCD, which yielded unsatisfactory C‐indices (0.642 and 0.633, respectively, in derivation and validation cohorts). This result is in line with recent studies validating the HFA‐ICOS score for stratifying CTRCD risk in HER2+ breast cancer [area under the curve (AUC): 0.58 and 0.643].^17^, ^18^ The possible explanation is that the HFA‐ICOS score is based on level of evidence B or C and only depends on clinical cardiovascular risk factors.^7^ This indicates the need to develop new biomarkers for the baseline risk stratification of CTRCD in breast cancer patients receiving anthracyclines.

The FD of ventricular trabeculae calculated based on CMR cine images is an important biomarker of cardiac function.^19^, ^20^ Previous studies have shown that the maximal apical FD of ventricular trabeculae is a predictor of cardiac adverse events in patients with pulmonary hypertension and hypertrophic cardiomyopathy.^14^, ^21^ Interestingly, we found that the maximal apical FD of left ventricular trabeculae was an ideal marker for baseline risk stratification of CTRCD in breast cancer patients receiving anthracyclines. The potential explanation may be that trabecular complexity represented by the FD is determined by cardiac genes and is an important biomarker of individual variation in cardiac efficiency.^12^ The maximal apical FD of left ventricular trabeculae can be calculated on the images of basic cine sequence in CMR.^22^ With short scanning time and no need for contrast agent,^23^ maximal apical FD of left ventricular trabeculae may be recommended for the risk stratification of CTRCD in breast cancer patients receiving anthracyclines. It is conducive to the implementation of the latest cardiac protective treatment.^24^

Our LVTFD‐based score classified 50.0% and 22.7% of participants with CTRCD as moderate‐ and high‐risk, whereas HFA‐ICOS score classified 31.8% and 18.2% of them as moderate‐ and high‐risk in the derivation cohort, and the results were similar in the validation cohort. This indicated that more CTRCD participants can be identified with medium‐high risk using LVTFD‐based score compared with medium‐high risk using HFA‐ICOS score. Possible explanations are as follows: First, the occurrence of CTRCD was attributable not only to clinical risk factors but also to the cardiac phenotype, which determines the heart's ability to tolerate adverse stimuli such as anthracyclines.^25^ LVTFD‐based score is calculated based on clinical risk factors including age, hypertension, previous CVD and CMR‐derived cardiac phenotype biomarker, and maximal apical FD of left ventricular trabecular, which is associated with risk of CVD.^12^, ^20^, ^26^ The HFA‐ICOS score is calculated only based on cardiovascular risk factors. Second, both the presence of CTRCD events and their timing were considered in the development of our new scoring system. This LVTFD‐based score classified 36.5% and 35.3% of participants without CTRCD as moderate‐risk, whereas HFA‐ICOS score classified 11.8% and 10.9% of them as moderate‐risk in derivation and validation cohorts, respectively. This indicated that participants with CTRCD classified as moderate‐risk by LVTFD‐based score maybe should undergo more frequent cardiotoxicity monitoring.

Previous studies showed that the incidence of CTRCD in breast cancer patients who received anthracyclines was 3%–20%.^27^, ^28^, ^29^ In our study, the incidence of CTRCD was 19.7%. There are several possible explanations. First, most patients come from remote rural areas with very low incomes. The anthracyclines received by most participants are doxorubicin, which is cheap and at high risk of cardiotoxicity. Second, the enrolled people are only from one region of a single country, and people from different countries and regions have different abilities to withstand adverse cardiac stimuli like anthracycline chemotherapy.

There were some limitations in this study. First, the enrolled population in our study is potentially skewed because all participants underwent CMR. Second, the LVTFD‐based score was derived from a single‐center study with a modest sample size. All participants were Asian with very low rate of baseline cardiovascular risk factors, differing considerably from western populations. Additional validations from multiple, international centers, and large samples are needed. Third, the median follow‐up is short in our study (18 months); long‐term follow‐up studies are needed to validate our scoring system.

In conclusion, LVTFD‐based score is an alternative metric for baseline risk stratification of CTRCD in breast cancer patients receiving anthracyclines. The LVTFD‐based score may be helpful for accurate baseline risk stratification of cardiotoxicity and for individualizing treatment strategies in breast cancer patients receiving anthracyclines.

## 4 MATERIALS AND METHODS

### 4.1 Study population

This prospective and single‐center study received approval from the local ethics committee and was conducted following the Declaration of Helsinki. All participants gave written informed consent.

Between January 2020 and March 2022, consecutive participants with breast cancer scheduled to receive anthracyclines and underwent CMR cine imaging were enrolled in this prospective study. The inclusion criteria were as follows: (i) pathologically confirmed female breast cancer; (ii) no history of anti‐tumor treatment before CMR examination. The exclusion criteria were as follows: (i) contraindication to CMR and (ii) serious artifacts of CMR.

All enrolled participants were randomly divided into the derivation or the validation cohort in a 3:2 ratio.

### 4.2 CMR protocol

CMR was performed on a 3.0‐T magnet (Ingenia, Philips Healthcare) with a 32‐channel coil array agent. Compressed sensing cine sequences in short‐axis, two‐chamber, three‐chamber, and four‐chamber views were performed with electrocardiogram‐gating and breath‐holding (8‐mm thick with a 10% gap). The detailed parameters of CMR imaging are summarized as follows: readout sequence; balanced steady‐state free precession; slice thickness/gap: 8 mm; repetition time: 3 ms; echo time: 1.48 ms; SENSE factor: 2; phase partial Fourier: off; average: 1; bandwidth: 1645 Hz; flip angle: 45°; field of view: 270 × 270 mm; Voxel size: 1.8 × 1.8 mm; calculated phases: 30.

### 4.3 CMR cine data analysis

CMR cine data were analyzed by two radiologists with 5 and 10 years of cardiac imaging experience blinded to the clinical and grouping information on cvi42 software version 5.16 (Circle Cardiovascular Imaging Inc.).

In the tissue tracking module, epicardial and endocardial contours were automatically generated and manually corrected if necessary. LVEF, LVEDV, LVESV, LVMASS, GLS, GCS, and GRS were measured. The GLS and GCS values are expressed as absolute numbers.

FD analysis was performed by the two radiologists mentioned above using the cvi42 software, prototype5.3.8. Endocardial contour was automatically traced at the end‐diastolic phase of every short‐axis slice and manually adjusted if necessary. Then, the FD values of each slice were automatically calculated. The left ventricular stack was split into apical and basal halves (Figure S1); the maximum and mean apical and basal FD and global FD were reported as a previous study (Figure S2).^14^ The global FD was defined as the mean value of FDs in all left ventricular slices. The mean apical or basal FD was defined as the mean value of all slices of the apex or base of the left ventricle. The maximum apical or basal FD was defined as the maximum value of all slices of the apex or of base the left ventricle.

### 4.4 CTRCD definition and monitoring

CTRCD was defined as ≥10% reduction in echocardiography measured LVEF to <53% according to the American Society of Echocardiography/European Association of Cardiovascular Imaging.^30^ For monitoring CTRCD, the cardiac troponin, natriuretic peptides, and echocardiography were performed every two cycles during anthracycline chemotherapy, every 3 months within 1 year after therapy completion, every 6 months with 1–2 years after therapy completion, and annually more than 2 years after therapy completion. All participants were followed for at least 1 year, the endpoint of follow‐up was April 2023. CTRCD‐free survival time was recorded from the start of anthracyclines to the occurrence of CTRCD or the endpoint of follow‐up.

### 4.5 Derivation of LVTFD‐based score

Clinical and CMR variables with *p* < 0.2 or clinically known CVD risk factors (e.g., age, body mass index, diabetes, hypertension, dyslipidemia, and smoking) regardless of *p* value in univariate analysis were entered into a multivariate Cox proportional hazards model analysis. Variables with *p* values < 0.10 in the multivariate analysis were then used to construct a risk score.^31^ The point of each covariate is equal to its coefficient divided by the coefficient with the smallest absolute value in the model and rounded to an integer. The LVTFD‐based score for a given individual was the total sum of points. The optimal cut‐off values for FD and the LVTFD‐based score for baseline risk stratification of CTRCD were calculated using X‐tile software (version 3.6.1; Yale University). Then, the participants were divided into low‐, moderate‐, and high‐risk subgroups based on the cut‐off value.

### 4.6 HFA‐ICOS score calculation

Moderate 1 and 2, high‐risk, and very high‐risk factors were defined according to HA‐ICOS score proforma. HFA‐ICOS score was divided into four risk subgroups as follows: low risk, no risk factors, or one Moderate 1 risk factor; moderate risk, moderate risk factors with a total of 2–4 points (Moderate 1 = 1 point; Moderate 2 = 2 points); high‐risk, moderate‐risk factors with a total of ≥5 points or any high‐risk factor and very high‐risk factor.^16^

### 4.7 Statistical analysis

The ICC was calculated for evaluating the reproducibility of continuous variables. The normality of data was tested with Shapiro–Wilk test. Mean and SD were used to present continuous variables with normal distribution. Median and IQR were used to present continuous variables with non‐normal distribution. Frequencies and proportions were used to present categorical variables. The two‐sample Student's *t*‐test or Mann–Whitney U test was used to compare continuous variables, and the chi‐square or Fisher exact test was used to compare categorical variables between participants with and without CTRCD. Univariable and multivariable Cox proportional hazard model was used to access the associated variables with CTRCD. The proportional hazards assumption test hold water. The performance of risk CTRCD was evaluated by Harrell's C‐statistic (C‐index) and was compared using Delong's test. CTRCD‐free survival in three different risk subgroups was compared using Kaplan–Meier curves and log‐rank test. Pairwise comparisons were performed using the Bonferroni correction, with a two‐tailed *p* value of <0.05/3 indicating a statistical difference. For the remaining statistics, two‐tailed *p* value of <0.05 indicated a statistical difference. Statistical analyses were performed in R (version 3.6.2; The R Foundation).

## AUTHOR CONTRIBUTIONS

*Conceptualization*: Hesong Shen, Jiuquan Zhang, and Qian Xu. *Methodology*: Chunrong Tu and Yangling Peng. *Investigation*: Zhiming Miao. *Statistical analyses*: Yuhang Xie and Rui Yang. *Writing—original draft*: Hesong Shen. *Writing—review and editing*: Jiuquan Zhang, Chunrong Tu, and Qian Xu. *Funding acquisition*: Jiuquan Zhang, Hesong Shen, and Chunrong Tu. Hesong Shen and Jiuquan Zhang had unrestricted access to all data. All authors agreed to submit the manuscript, read, and approve the final draft and take full responsibility of its content. Jiuquan Zhang had final responsibility for the decision to submit for publication.

## ETHICS STATEMENT

This clinical study was approved by the ethics committee of Chongqing University Cancer Hospital (CZLS2020238‐A).

## CONFLICT OF INTEREST STATEMENT

The authors declare no conflicts of interest.

## Supporting information



Supporting Information

## Data Availability

The data that support the findings of this study are available from the corresponding author upon reasonable request.
