# Supplementary material for: Left ventricular trabecular complexity for risk stratification of cancer therapy–related cardiac dysfunction in breast cancer
Source: MedComm (2020). 2025 Jan 2;6(1):e70004. doi: 10.1002/mco2.70004 (PMC11695210; doi:10.1002/mco2.70004)

**Title Page**

**Title:** Left ventricular trabecular complexity for risk stratification of cancer therapy-related cardiac dysfunction in breast cancer

**Runing title**: CMR in predicting cardiotoxicity in breast cancer

Hesong Shen^1^, MD, Qian Xu^2^, MD, ChunrongTu^1^, MD, Yangling Peng^1^, MD, Yuhang Xie^1^, MD, Zhiming Miao^1^, BS, Rui Yang^1^, Jiuquan Zhang^1*^, PhD

^1^Department of Radiology, Chongqing University Cancer Hospital & Chongqing

Cancer Institute & Chongqing Cancer Hospital, Chongqing, China.

^2^School of Medicine, Chongqing University, Chongqing, China.

***Correspondence Address:**

Jiuquan Zhang, Department of Radiology, Chongqing University Cancer Hospital & Chongqing Cancer Institute & Chongqing Cancer Hospital, Chongqing 400030, P. R. China

Tel: +86-23-65079339.

Fax: +86-23-65079339

E-mail: zhangjq_radiol@foxmail.com

**Supplemental Material**

**Supplementary Tables**

**Table S1** Intra- and inter-observer reproducibility of these parameters derived from cardiac magnetic resonance cine images

|  | Intraclass coefficient correlation (95 % CI) | |
| --- | --- | --- |
|  | Intra-observer | Interobserver |
| LVEF | 0.928 (0.823-0.962) | 0.905 (0.803-0.947) |
| LVEDV | 0.925 (0.795-0.982) | 0.896 (0.805-0.963) |
| LVESV | 0.915 (0.806-0.931) | 0.909 (0.790-0.928) |
| LVMASS | 0.957 (0.879-0.980) | 0.940 (0.861-0.977) |
| GLS | 0.897 (0.875-0.915) | 0.846 (0.765-0.894) |
| GCS | 0.876 (0.851-0.898) | 0.895 (0.841-0.926) |
| GRS | 0.867 (0.814-0.902) | 0.859 (0.810-0.890) |
| Global FD | 0.935 (0.920-0.947) | 0.891 (0.866-0.911) |
| Maximal basal FD | 0.931 (0.915-0.944) | 0.888 (0.863-0.909) |
| Mean basal FD | 0.937 (0.923-0.949) | 0.886 (0.860-0.907) |
| Maximal apical FD | 0.936 (0.921-0.948) | 0.898 (0.874-0.916) |
| Mean apical FD | 0.924 (0.907-0.938) | 0.895 (0.872-0.915) |

Abbreviations: FD, fractal dimension; GLS, global longitudinal strain; GCS, global circumferential strain; GRS, global radial strain; LVEF, left ventricular ejection fraction; LVEDV, left ventricular end-diastolic volume; LVESV, left ventricular end-systolic volume; LVMASS, left ventricular mass.

**Table S2** Correlations between left ventricular FD and ventricular function and mass parameters in derivation cohort

|  | Global FD | | Maximal basal FD | | Mean basal FD | | Maximal apical FD | | Mean apical FD | |
| --- | --- | --- | --- | --- | --- | --- | --- | --- | --- | --- |
|  | *r* | *P* | *r* | *P* | *r* | *P* | *r* | *P* | *r* | *P* |
| LVEF | -0.046 | 0.49 | -0.042 | 0.54 | -0.055 | 0.41 | -0.042 | 0.54 | -0.045 | 0.51 |
| LVEDV | 0.049 | 0.47 | 0.052 | 0.44 | 0.048 | 0.48 | 0.055 | 0.42 | 0.048 | 0.48 |
| LVESV | 0.067 | 0.32 | 0.062 | 0.36 | 0.064 | 0.34 | 0.069 | 0.31 | 0.061 | 0.36 |
| LVMASS | -0.094 | 0.16 | -0.093 | 0.17 | -0.103 | 0.13 | -0.089 | 0.19 | -0.096 | 0.16 |
| GLS | 0.045 | 0.51 | 0.057 | 0.40 | 0.055 | 0.42 | 0.065 | 0.33 | 0.056 | 0.41 |
| GRS | -0.111 | 0.098 | -0.122 | 0.070 | -0.111 | 0.10 | -0.117 | 0.081 | -0.114 | 0.091 |
| GCS | -0.062 | 0.35 | -0.062 | 0.36 | -0.069 | 0.31 | -0.062 | 0.36 | -0.065 | 0.33 |

Abbreviations: FD, fractal dimension; GLS, global longitudinal strain; GCS, global circumferential strain; GRS, global radial strain; LVEF, left ventricular ejection fraction; LVEDV, left ventricular end-diastolic volume; LVESV, left ventricular end-systolic volume; LVMASS, left ventricular mass.

**Table S3** Correlations between left ventricular FD and ventricular function and mass parameters in validation cohort

|  | Global FD | | Maximal basal FD | | Mean basal FD | | Maximal apical FD | | Mean apical FD | |
| --- | --- | --- | --- | --- | --- | --- | --- | --- | --- | --- |
|  | r | *P* | r | *P* | r | *P* | r | *P* | r | *P* |
| LVEF | -0.068 | 0.41 | -0.057 | 0.49 | -0.063 | 0.44 | -0.059 | 0.47 | -0.056 | 0.50 |
| LVEDV | 0.004 | 0.96 | -0.001 | 0.99 | -0.002 | 0.98 | -0.005 | 0.96 | -0.001 | 0.99 |
| LVESV | 0.047 | 0.57 | 0.035 | 0.67 | 0.041 | 0.62 | 0.039 | 0.64 | 0.036 | 0.66 |
| LVMASS | -0.044 | 0.59 | -0.038 | 0.65 | -0.035 | 0.67 | -0.033 | 0.69 | -0.039 | 0.64 |
| GLS | -0.011 | 0.89 | 0.003 | 0.97 | 0.013 | 0.87 | 0.024 | 0.78 | 0.009 | 0.91 |
| GCS | 0.089 | 0.28 | 0.082 | 0.32 | 0.086 | 0.30 | 0.084 | 0.31 | 0.082 | 0.32 |
| GRS | -0.093 | 0.26 | -0.104 | 0.21 | -0.092 | 0.26 | -0.096 | 0.25 | -0.098 | 0.24 |

Abbreviations: FD, fractal dimension; GLS, global longitudinal strain; GCS, global circumferential strain; GRS, global radial strain; LVEF, left ventricular ejection fraction; LVEDV, left ventricular end-diastolic volume; LVESV, left ventricular end-systolic volume; LVMASS, left ventricular mass.

**Figure S1** **Left ventricular slices on the four-chamber cine image.** Left ventricle including nine slices was divided into apical and basal halves.


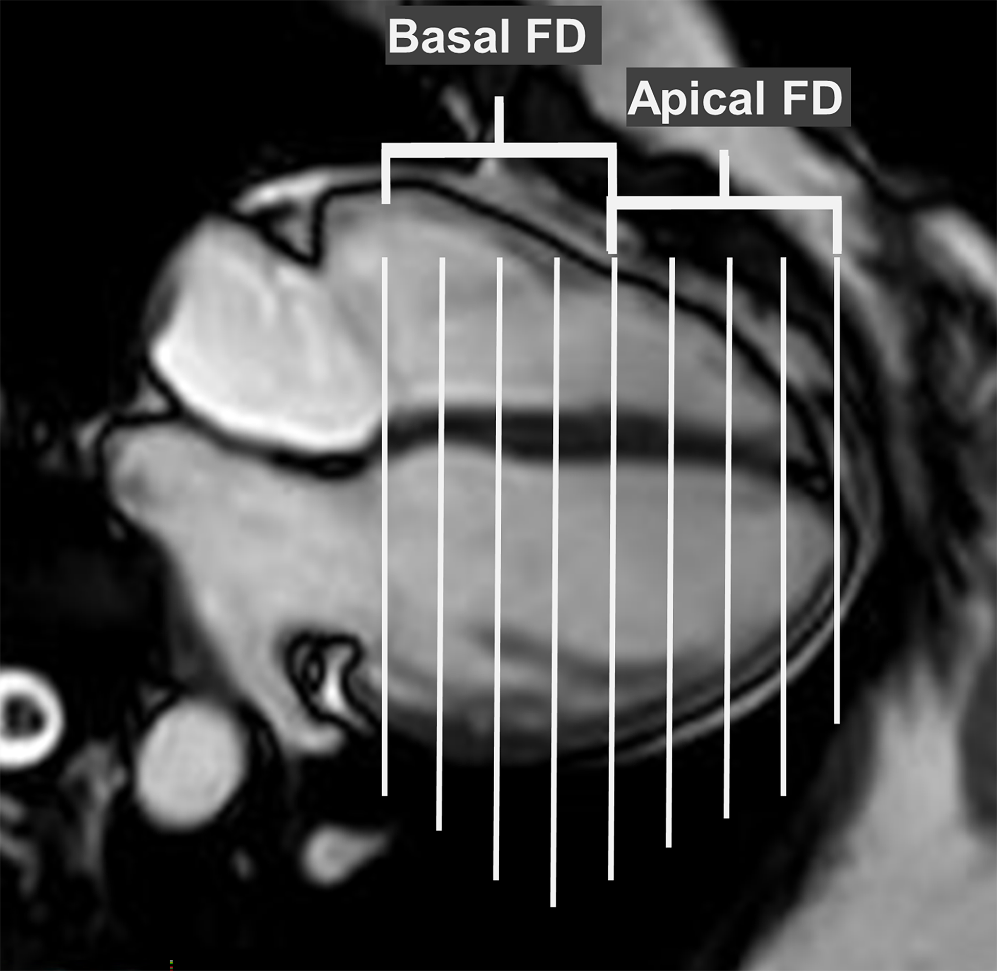


**Figure S2** **Diagram of FD calculation.** The global FD was defined as the mean value of FDs in all left ventricular slices (slices1–9). The mean apical or basal FD was defined as the mean value of all slices of the apex or base of the left ventricle (slices 1–5 or 5–9). The maximum apical or basal FD was defined as the maximum value of all slices of the apex or of base the left ventricle (slices 1–5 or 5–9). FD, fractal dimension.


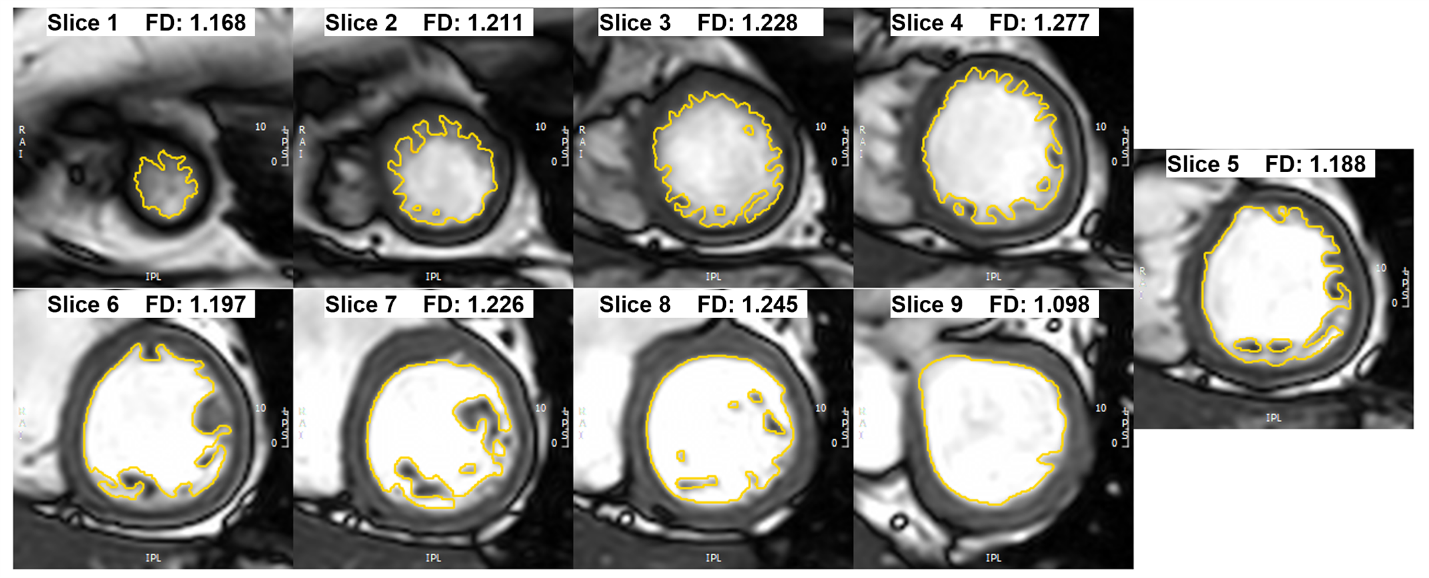

Supplement: Supplementary file 1 — Supporting Information [file MCO2-6-e70004-s001.docx]
